# Multi-Objective Optimization Design and Impact Protection Efficacy of Locally Reinforced P-TPMS Forehead Helmet Liner

**DOI:** 10.3390/ma19122571

**Published:** 2026-06-14

**Authors:** Bin Yang, Hao Feng, Xin Li, Peng Zhang, Li Li, Xinyu Wei, Zongchen Su, Qi Jin, Jiawei Zhang, Jianhao Zhang

**Affiliations:** School of Transportation Engineering, Nanjing Institute of Technology, Nanjing 211167, China; y00450240411@njit.edu.cn (H.F.); y00450240142@njit.edu.cn (P.Z.); y00450240321@njit.edu.cn (L.L.); y00450240338@njit.edu.cn (X.W.); y00452501224@njit.edu.cn (Z.S.); y00452511101@njit.edu.cn (Q.J.); y00452511117@njit.edu.cn (J.Z.); y00452511118@njit.edu.cn (J.Z.)

**Keywords:** triply periodic minimal surface, helmet liner, impact protection, finite element analysis, response surface optimization, head injury criterion

## Abstract

The objective of this study is to mitigate the bottom-out failure and improve the energy absorption of conventional helmet liners during high-energy impacts, thereby reducing the risk of head injuries. To this end, a locally reinforced Primitive-type triply periodic minimal surface (P-TPMS) energy-absorbing liner is proposed for the helmet forehead region, which facilitates progressive energy dissipation through layer-by-layer buckling deformation. A finite element model of a helmet–head coupling was created based on a previously verified high-fidelity head model and subsequently validated against the ECE 22.06 standard drop-test methodology. Three critical design parameters—outer protective layer thickness, triply periodic minimal surface (TPMS) unit cell size, and wall thickness—were optimized employing the Box–Behnken Design (BBD) response surface methodology, resulting in quadratic regression models for the head injury criteria (*HIC*) and peak linear acceleration (*PLA*) with good fit ($R^{2}$ > 0.97). Optimal parameter combinations were established using multi-objective optimization, with protective efficacy carefully assessed from both head dynamic response and biomechanical response perspectives. The ideal P-TPMS liner possesses an outer protective layer thickness of 14.95 mm, a TPMS unit cell size of 12.23 mm, and a wall thickness of 3.93 mm. Compared to the traditional expanded polystyrene (EPS) liner, the optimized P-TPMS liner significantly reduces *HIC* (by ∼16%) and *PLA* (by ∼14%) while extending the impact duration. More critically, it transitions both intracranial pressure and brain tissue strain below their respective clinical injury thresholds, substantially lowering the risks of skull fracture and mild traumatic brain injury (mTBI). The P-TPMS construction facilitates continuous energy dissipation during impacts via incremental layer-by-layer buckling deformation, hence extending impact duration and markedly improving helmet protective efficacy. These findings offer theoretical foundations and technical direction for the creation of localized heterogeneous liner designs in advanced high-performance helmets, although the results are limited to frontal flat-anvil impact conditions.

## 1. Introduction

Road traffic collisions have emerged as a significant concern in the domain of global public health. The World Health Organization’s *Global Status Report on Road Safety 2023* reveals that almost 1.19 million individuals died in road traffic accidents globally in 2021, with operators of two- and three-wheeled vehicles representing 21% of these fatalities [1]. Head injuries constitute the predominant cause of mortality and disability in road traffic collisions, with systematic reviews affirming their status as the most prevalent and lethal injury type in motorcycle accidents [2]. The shell and liner of a helmet work together to spread out impact forces and soak up energy. This reduces the acceleration that is passed on to the head and lowers the risk of brain tissue damage [3]. The design of the energy-absorbing liner and the impact location significantly influence the protective effectiveness of helmets. Epidemiological studies indicate that the forehead is a common site for helmet impacts, with empirical analyses showing that forehead impacts account for approximately 19% of all incidents [4]. A meta-analysis of 1809 head impacts further confirmed that the forehead and lateral regions are the predominant impact areas [5]. More importantly, frontal impact is closely associated with the development of subdural hematomas, and the resulting brain injury is often more severe [6]. Consequently, reinforcing solutions targeting the forehead area warrant investigation as a practical approach to improving helmet protective efficacy.

A conventional motorcycle helmet comprises a rigid outer shell—commonly fabricated from polycarbonate, fiberglass composites, carbon fiber, or occasionally aluminum, selected for their ability to distribute impact forces and resist fracture—and an energy-absorbing inner liner extensively utilizing EPS foam due to its lightweight nature, affordability, and effective energy absorption characteristics. The helmet investigated in this study employs an acrylonitrile butadiene styrene (ABS) resin shell, which is widely adopted in mid-range motorcycle helmets for its balanced impact resistance and manufacturing practicality. During an impact event, the outer shell and inner liner function synergistically: the shell distributes the localized impact force over a larger area through elastic flexure and, in severe cases, controlled cracking, thereby reducing the force concentration transmitted to the liner; simultaneously, the EPS foam liner undergoes controlled plastic deformation and progressive compression, converting kinetic energy into permanent structural deformation and dissipating it through cell-wall buckling and collapse. The stress–strain curve of EPS foam demonstrates a conventional three-stage pattern: linear elasticity, a yield plateau, and densification. During high-energy impacts, the plateau stress stage is notably short, causing the material to swiftly transition into the densification stage, resulting in a rapid increase in stress and a sudden surge in acceleration, referred to as "bottoming-out." This immediate densification mechanism, dictated by the material’s constitutive properties, establishes a physical limit on the energy absorption capacity of EPS that is challenging to surpass [7]. Consequently, the development of novel liner constructions incorporating sophisticated energy dissipation mechanisms has become the principal strategy for rectifying existing protective inadequacies [8]. Moreover, conventional EPS liners demonstrate considerable deficiencies in mitigating the risk of traumatic brain injury (TBI), particularly in high-impact scenarios where energy absorption is critical for protecting the brain from trauma [9].

Recently, TPMS designs have provided novel insights into helmet liner design owing to their distinctive geometric qualities and superior mechanical attributes [10]. TPMS refers to a category of periodic surface structures characterized by zero mean curvature, commonly observed in natural systems like biological membranes and butterfly wing scales [11]. Their statistically manageable geometric configurations and superior physical and mechanical characteristics indicate significant potential in metamaterials and meta-composites [12]. A systematic evaluation reveals that TPMS structures have a gradual, layer-by-layer buckling deformation under compression, showcasing markedly enhanced energy absorption relative to traditional foam materials [13]. The Schwarz Primitive (P-type) structure, among the different TPMS types, has garnered significant attention for its distinctive mechanical response attributes. Maskery et al. [14] utilized a hybrid experimental and numerical simulation methodology to thoroughly examine the mechanical properties of diverse TPMS lattice configurations. The relative elastic modulus of the P-type lattice exceeds double that of the Gyroid and Diamond types. The deformation process demonstrates a clear tensile-buckling-dominated mode; in contrast to bending-dominated structures, this mode sustains a prolonged plateau stress area, resulting in enhanced energy dissipation at high-strain-rate impact scenarios. Subsequent study reveals that gradient P-type structures display a layer-by-layer progressive collapse tendency under compression, achieving a total energy absorption of 2.47 MJ/m^3^, hence exhibiting superior energy absorption capabilities [15].

Thus far, the implementation of TPMS structures in protective equipment has achieved preliminary advancements. Liu et al. [16] created four types of biomimetic TPMS heterogeneous structures, inspired by the architecture of femoral trabeculae, and applied them to bicycle helmets, revealing that stress-adaptive porous components exhibit exceptional modeling flexibility and mechanical strength. The incorporation of heterogeneous components augmented the strength of the original structure by over 25% and improved the total energy absorption properties by more than 23.5%. Moreover, evaluation studies utilizing high-fidelity finite element models of the head have shown that the optimized design of helmet liners necessitates thorough consideration of microstructural characteristics, including strain distribution in brain tissue [17] and the orientation of white matter fiber bundles [18]; dependence solely on kinematic indicators complicates a comprehensive assessment of the risk of TBI.

It is worth noting that TPMS geometrical features can be exploited in multiple ways beyond complete lattice replacement. Recent studies have demonstrated that TPMS-based geometrical modifications can be strategically integrated into conventional cellular structures to enhance their mechanical performance. For instance, Iandiorio et al. [19] showed that introducing TPMS-inspired fillet shapes into simple cubic lattices significantly improved the strength-to-weight ratio under both static and dynamic compression. The TPMS-type fillet shapes induced a triaxial stress state that enhanced mechanical strength compared to fillet-free lattices. This finding is conceptually important because it demonstrates that TPMS geometries do not necessarily need to replace an entire lattice topology; rather, they can be deployed locally to improve stress transfer and delay failure initiation. This perspective supports the engineering rationale of the present study, which focuses TPMS reinforcement on the high-impact forehead region rather than replacing the entire helmet liner. Such a localized approach targets structural functionality where it is statistically most needed while reducing manufacturing complexity compared to full-liner replacement strategies.

Nevertheless, current research exhibits several gaps: firstly, the majority of investigations into TPMS helmet liners utilize a complete liner replacement approach, which neglects localized heterogeneous reinforcement designs for the high-impact forehead area, thereby complicating the attainment of an optimal equilibrium between protective efficacy and manufacturing efficiency; secondly, the mechanisms through which TPMS structural parameters (outer protective layer thickness, TPMS unit cell size, and wall thickness) affect helmet protective performance are not well understood, and there is an absence of systematic multi-parameter coupled optimization studies. Thirdly, current research primarily employs a singular dynamic metric (e.g., *HIC*) for performance evaluation, failing to provide a thorough assessment of biomechanical indicators such as intracranial pressure and brain tissue strain, thereby complicating the elucidation of the protective mechanisms of TPMS liners against TBI.

This study proposes a forehead-localized reinforced helmet liner utilizing a P-TPMS structure and systematically investigates its impact protection performance through finite element simulation and response surface optimization. The contributions of this work are threefold: (1) proposing a localized reinforcement strategy that balances protective efficacy with manufacturing practicality, addressing a gap in existing TPMS helmet design approaches that predominantly focus on full-liner replacement; (2) systematically integrating finite element analysis with Box–Behnken response surface methodology for multi-parameter optimization of TPMS-based protective structures; and (3) establishing a comprehensive evaluation framework encompassing both kinematic metrics (*HIC*, *PLA*, skull fracture correlate (*SFC*)) and biomechanical metrics (peak intracranial pressure (*ICP*), maximum principal strain (*MPS*)), Abbreviated Injury Scale score of 2 or greater (AIS2+) for helmet liner assessment. This work provides a reference for the design of spatially heterogeneous liners in protective equipment.

## 2. Construction and Validation of the Finite Element Model

### 2.1. Finite Element Model of the Head

This research employed the 50th percentile human head finite element model established and validated by Tse et al. [20]. This model was developed from a survey and statistical analysis of Asian anthropometric characteristics, utilizing multimodal medical imaging scans of Singaporean Chinese volunteers who met the 50th percentile criteria, yielding a high-fidelity head finite element model with intricate anatomical structures. The original study methodically delineated its geometric architecture, meshing, and material qualities. The model has been further tested and utilized in several biomechanical studies of head traumas [21,22,23], confirming its reliability.

**Figure 1:**
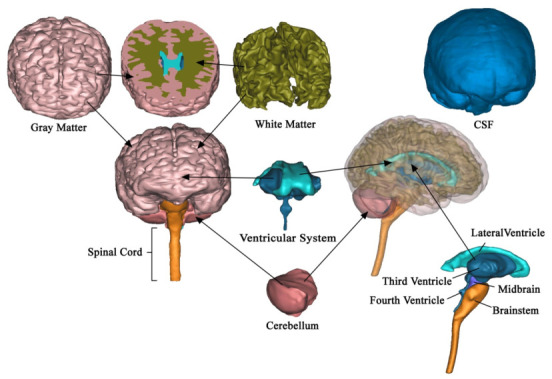
Three-dimensional Geometric Model of the Head [24].

The geometric construction of the model relies on multimodal picture fusion technology. Cranial geometric data was obtained from 460 axial CT images (in-plane resolution 512 × 512 pixels, pixel size 0.488 mm, slice thickness 1.0 mm); geometric data on brain tissue was acquired from MRI images (in-plane resolution 1659 × 962 pixels, pixel size 0.500 mm, slice thickness 4.0 mm). After registering and merging the CT and MRI data, the information was uploaded into Mimics v14.0 (Materialise, Leuven, Belgium) for three-dimensional reconstruction and segmentation. The skull was meticulously divided into 14 osseous segments, including 8 cranial bones (1 frontal, 1 occipital, 1 ethmoid, 1 sphenoid, 2 parietal, and 2 temporal bones) and 6 facial bones (1 palatine, 1 maxillary, 1 mandibular, 1 nasal, and 2 zygomatic bones), each delineated by its respective sutures; the model preserved air-filled sinus cavities, including the frontal, sphenoid, and maxillary sinuses. The brain tissue was divided into the cerebrum and cerebellum, encased by a layer of cerebrospinal fluid. Figure 1 illustrates the reconstructed head geometry.

**Figure 2:**
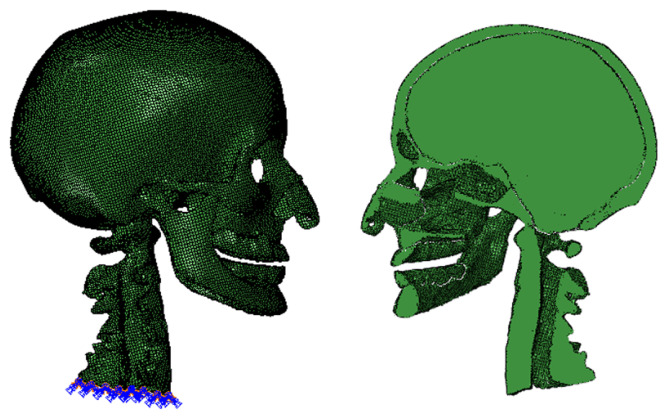
Finite Element Model of the Head.

After remeshing the surface mesh with the 3-matic module, mesh repair and optimization were conducted in HyperMesh v11.0 (Altair HyperWorks, Troy, MI, USA). The completed finite element model consisted of 327,536 nodes and 1,173,039 linear tetrahedral elements, featuring an average edge length of roughly 1.57 mm and an aspect ratio of 1.61. The cerebrospinal fluid layer was represented by approximately 2–3 layers of solid elements to emulate the fluid interface between the skull and brain tissue; this thickness was validated through mesh convergence analysis and effectively captured the relative motion between the skull and brain. Figure 2 illustrates the finite element model.

Concerning material properties, brain tissue is represented by a linear viscoelastic model integrated with large-deformation theory to effectively represent its rate-dependent response and non-linear deformation traits under impact loads; cerebrospinal fluid is characterized as a linear elastic solid with low stiffness and low shear modulus to emulate its liquid cushioning properties; and the skull and other skeletal tissues are modeled using linear elastic, isotropic material frameworks. The specific material properties for each tissue are enumerated in Table 1.

Model validation is an essential process for confirming the dependability of simulation outcomes. Upon modifying the density of each element of this head model, the overall weight adheres to human statistical norms [38]. The biomechanical validity of the model has been substantiated through three sets of cadaver experiments [39,40,41]: by juxtaposing the *ICP* time-course curves and intracranial relative displacement derived from the simulation with experimentally obtained values, the findings demonstrate strong concordance in peak values, trends, and decay characteristics, thereby affirming the model’s reliability in forecasting head impact responses [20].

### 2.2. Finite Element Model of the Helmet

Motorcycle helmets are composed of elements including the shell, energy-absorbing liner, comfort liner, adjustable straps, and visor. Their protective efficacy—especially their energy-absorption capability—largely relies on the integrated design of the shell and the energy-absorbing liner [42]. The commercial motorcycle helmet shell selected for this study is made of ABS resin, and the energy-absorbing lining is made of EPS foam.

Due to the pronounced geometric non-linearity of motorcycle helmets, conventional computer-aided design (CAD) software encounters difficulties in precisely modeling their intricate surfaces. This work utilizes a reverse engineering methodology to develop the finite element model of the helmet. The detailed technique is outlined as follows:

#### 2.2.1. Model Simplification

In the reverse engineering process, components with little influence on energy absorption—such as the visor rotation mechanism, ventilation system, cosmetic elements, and comfort liner—were omitted to streamline the computational model [43]. Ultimately, three fundamental components were preserved: the shell, the energy-absorbing liner, and the retaining straps.

#### 2.2.2. 3D Scanning

**Figure 3:**
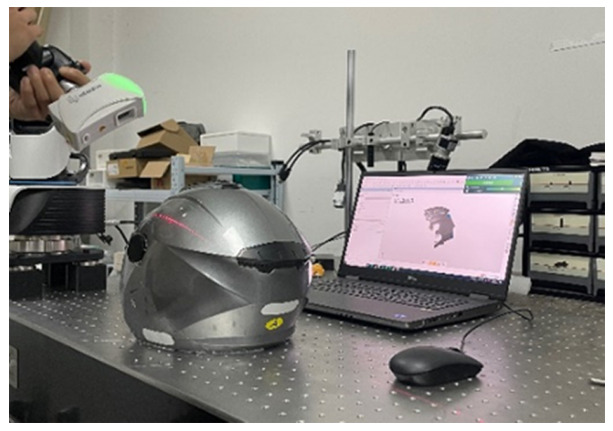
Line-structured light scanning.

A six-axis articulated arm coordinate measuring machine, fitted with a HEXAGON HP-L-8.9 T2 (Hexagon AB, Stockholm, Sweden) scanning probe, conducted structured light scanning on the helmet’s primary structure (shown in Figure 3). Subsequent to denoising and smoothing the obtained point cloud data, it was transformed into a triangular mesh model.

#### 2.2.3. Construction of Geometric Models and Generation of Meshes

**Figure 4:**
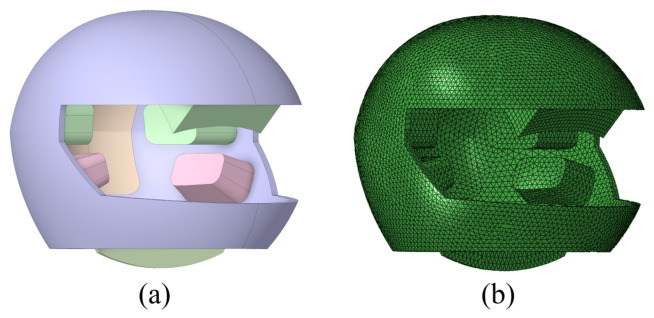
Helmet Model: (**a**) helmet geometric model; (**b**) helmet finite element model.

The processed model was loaded into Catia V5 R21 (Dassault Systèmes, Vélizy-Villacoublay, France), and the geometric model was recreated using operations including surface generation, fitting, and thickening. The outer shell and energy-absorbing liner preserve the shapes acquired by scanning technology, guaranteeing a snug fit of the inner surface of the shell against the energy-absorbing liner during assembly, thus effectively replicating the structural integrity of the original helmet [44]. Figure 4a displays the geometric model of the reconstructed helmet.

The geometric model was imported into HyperMesh v11.0 for meshing and quality enhancement; the resultant finite element model of the helmet is depicted in Figure 4b. Detailed information on element types, mesh sizes, and contact definitions is provided in Section 2.2.5.

#### 2.2.4. Material Properties

**Figure 5:**
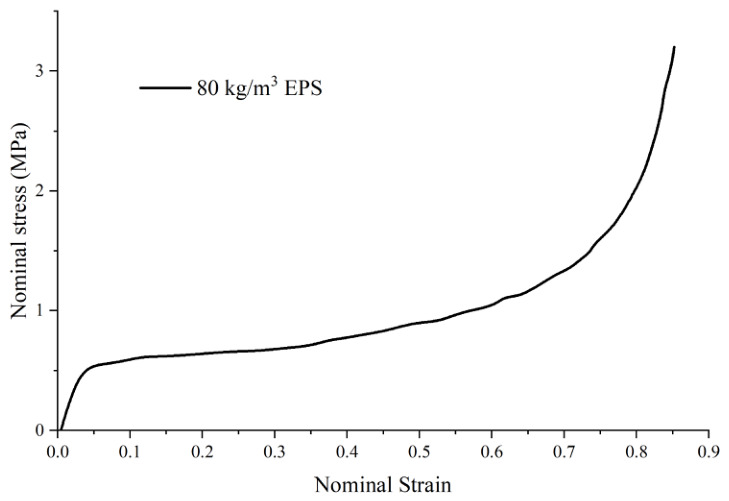
Stress–strain curves for 80 kg/m^3^ EPS foam liner material.

Table 2 delineates the material specifications for the helmet shell (ABS resin) and straps, which are modeled as linearly elastic materials. For the EPS foam liner, a crushable foam plasticity model in Abaqus/Explicit v2022 (SIMULIA, Providence, RI, USA) was utilized to characterize its crushing behavior under impact loads. The stress–strain curve was obtained from the experimental calibration results of Cui et al. [45] for EPS foam with a density of 80 kg/m^3^, as illustrated in Figure 5.

For the TPMS forehead liner structure, the same EPS material model was adopted in this preliminary study. This simplification is based on the following considerations: (1) the primary objective of this study is to investigate the structural performance enhancement through TPMS geometry optimization rather than material innovation; (2) using the same material model for both the original EPS liner and the TPMS insert enables a fair comparison of structural effects while controlling for material variables; and (3) this approach allows for isolation of the geometric contribution to protective performance improvement. In other words, the contribution of the P-TPMS model in this study lies in its geometric architecture—specifically, the layer-by-layer buckling deformation mechanism and the localized reinforcement strategy—rather than in the material constitutive response. However, we acknowledge that this simplification has limitations, as the mechanical behavior of thin-walled TPMS structures fabricated by additive manufacturing may differ from bulk EPS foam due to scale effects and manufacturing process characteristics. Future studies should incorporate material models calibrated from actual additively manufactured polymer specimens.

#### 2.2.5. Finite Element Modelling Details

To ensure reproducibility and provide comprehensive documentation of the numerical model, the finite element modelling details are summarized in this section. Table 3 presents the element types, mesh sizes, and contact definitions for all components in the helmet–head coupled model.

Shell and straps were meshed using 8-node reduced integration hexahedral elements (C3D8R) with a nominal element size of 5 mm. The EPS liner and TPMS forehead liner were meshed using linear tetrahedral elements (C3D4) with a nominal element size of 3 mm to better capture the complex geometry of the TPMS lattice structure. The anvil was modeled as a rigid body using hexahedral elements.

Regarding contact definitions, surface-to-surface contact was defined between the head and liner interfaces to allow for relative sliding and separation during impact. The friction coefficient between the head and liner was set to 0.3, following the ECE 22.06 standard specification for oblique impact testing [47]. The EPS liner, TPMS forehead liner, and straps were connected to the inner surface of the shell using tied constraints, simulating the adhesive bonding in actual helmet construction.

Hourglass control was implemented using the default stiffness-based formulation in Abaqus/Explicit v2022 for reduced integration elements (C3D8R), with no additional parameters modified from the default settings. No mass scaling was applied in the simulations. The stable time increment was determined by the smallest element size and the material wave speed, resulting in an average time increment of approximately 7 × 10^−8^ s. The total simulation duration was 20 ms, with output intervals of 0.1 ms for acceleration and displacement data extraction.

Mesh convergence analysis was conducted to verify the numerical solution accuracy for the helmet–head coupled model with the original EPS liner. Three mesh densities were examined for the EPS liner: coarse (5 mm nominal element size, approximately 28,000 elements), medium (3 mm nominal element size, approximately 72,000 elements), and fine (2 mm nominal element size, approximately 165,000 elements). The head model mesh remained unchanged across all cases. Table 4 presents the convergence results for global metrics (*HIC* and *PLA*).

The results indicate that coarser meshes yield higher *HIC* and *PLA* values, which is attributed to numerical stiffness enhancement in under-resolved discretizations. As the mesh is refined, both metrics decrease and approach convergence, with variations of 1.4% for *HIC* and 2.0% for *PLA* between medium and fine meshes. Considering the balance between computational accuracy and efficiency, the medium mesh (3 mm) was selected for all subsequent analyses. The same mesh strategy was applied to the TPMS forehead liner model to ensure consistent numerical accuracy across all simulations.

### 2.3. Validation of the Helmet–Head Coupling Model

**Figure 6:**
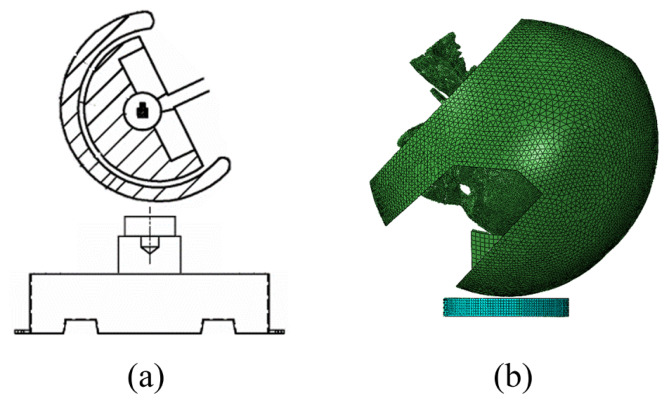
Comparison of helmet drop tests: (**a**) sample standard; (**b**) finite element simulation.

This work performed simulation-based validation of drop tests to verify the reliability of the helmet–head coupling model, adhering to the ECE 22.06 standard [47]. Figure 6a illustrates the physical test configuration: a metal headform was securely affixed to the helmet, which was elevated to a height of 2.9 m above a rigid flat anvil and subsequently released, resulting in the helmet impacting the anvil at an approximate velocity of 7.5 m/s. In the simulation, the helmet–head coupled model impacted the stationary flat anvil at an identical initial velocity of 7.5 m/s, whereas a vertical downward gravitational acceleration of −9.8 m/s^2^ was exerted, as illustrated in Figure 6b.

**Figure 7:**
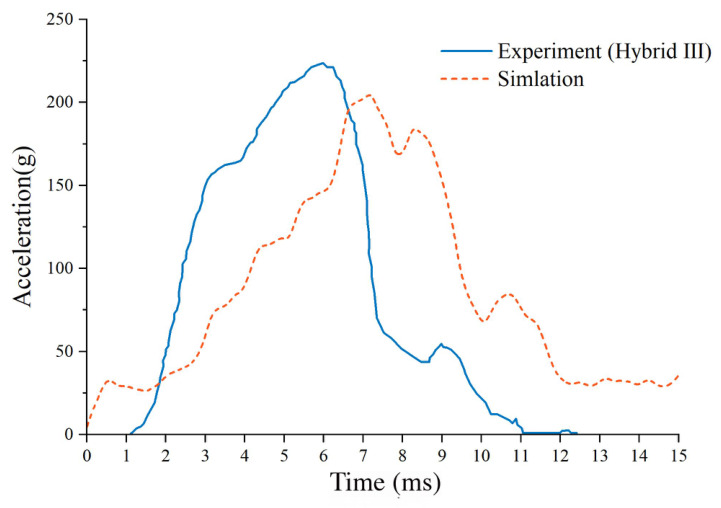
Comparison of experimental and simulated acceleration curves.

This work performed flat anvil impact validation at the forehead position; the experimental results [48] and simulation results for the acceleration of the head’s center of mass, both defined by gravitational acceleration g, are presented in Figure 7. The simulation results exhibit strong alignment with the Hybrid III dummy test data regarding reaction trends. Quantitative analysis based on digitized data extraction showed a peak acceleration of approximately 232.1 g for the experiment and 212.6 g for the simulation, yielding a relative error of approximately 8.4%. The peak time lag was approximately 1.40 ms. The correlation coefficient between the two curves increased from 0.41 (original) to 0.94 (after time-shift compensation), indicating good agreement in the overall waveform shape despite the phase lag. Additionally, the *HIC* value was calculated for both the experimental and simulated acceleration curves. The experimental *HIC* was approximately 1850, while the simulated *HIC* was 1634.62, yielding a relative error of approximately 11.6%. This *HIC* comparison provides a more comprehensive assessment of the model’s predictive accuracy for cumulative injury metrics.

The acceleration time history curve can be examined in three phases: during the loading phase from 0 to 7 ms, the acceleration curve consistently ascends, reflecting the impact compression process of the head–helmet system. Upon the transfer of impact load to the helmet, the energy-absorbing liner progressively compresses, transforming kinetic energy into structural deformation energy, with the system attaining peak acceleration at approximately 6–7 ms. The experimental peak reached approximately 220 g, whereas the simulation results were marginally lower and demonstrated some delay. Subsequent to the peak, the system transitioned into the unloading and rebound phase, characterized by a rapid fall in acceleration; the experimental curve displayed a pronounced decline, with a little sub-peak emerging at 8–9 ms, indicative of localized rebound and secondary contact phenomena. Conversely, the simulation curve exhibited a gradual fall, with distinct oscillatory traits during the mid-to-late phases.

The discrepancies between the experimental and simulation results are predominantly evident in peak lag and response variations, attributable to: (1) a negligible initial gap between the head and the liner in the simulation model, resulting in supplementary contact effects; (2) intrinsic differences in structural details and material properties between the finite element head model and the physical Hybrid III dummy, causing deviations in peak and residual acceleration.

The simulation findings capture the peak acceleration range and overall decay pattern with acceptable accuracy, demonstrating that the helmet–head coupling model is reasonably reliable for predicting head dynamic responses. It is important to distinguish between two aspects of validation: the present validation applies only to the general helmet–head coupled model with the original EPS liner, not to the proposed TPMS forehead liner. The TPMS liner design has not been validated through physical experiments, and its predictive accuracy relies on the assumption that the validated helmet–head model can reasonably represent head dynamic responses under modified liner configurations. Future studies should include physical impact tests of TPMS liners to verify the simulation predictions. Injury assessment indicates that peak head acceleration and its duration directly influence the *HIC* magnitude; the reasonable prediction of acceleration time history through simulation in this study implies that the model is suitable for head injury risk assessment within the acknowledged limitations.

## 3. Design and Improvement of the TPMS Forehead Helmet Liner

### 3.1. Design of the TPMS Forehead Helmet Liner

**Figure 8:**
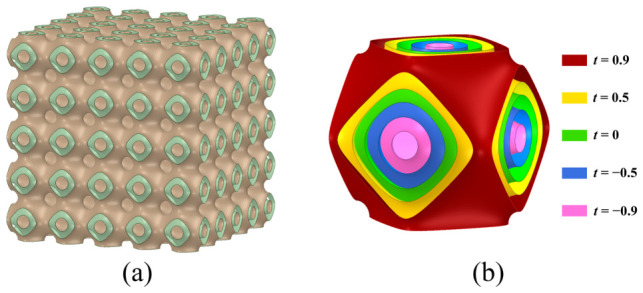
P-TPMS structure: (**a**) typical array structure; (**b**) P-TPMS lattice unit structure with different *t* values.

The TPMS structure, characterized by its smooth, continuous surface topology and high pore connectivity, facilitates efficient energy absorption and stress distribution, rendering it an optimal candidate for protective liners. This research selects the P-type arrangement as the energy-absorbing unit for the forehead liner, as seen in Figure 8a. This selection is based on the following considerations from the literature. Maskery et al. [14] conducted a systematic comparative study on the mechanical properties of various TPMS lattice configurations (Primitive, Gyroid, and Diamond) fabricated by polymer additive manufacturing. Their results demonstrated that the P-type lattice exhibits a relative elastic modulus more than twice that of Gyroid and Diamond types at comparable relative densities. More importantly, the deformation mode of P-type structures under compression shows a clear tensile-buckling-dominated behavior, which sustains a prolonged plateau stress region compared to bending-dominated structures, resulting in enhanced energy dissipation under high-strain-rate impact scenarios. Furthermore, Liu et al. [16] developed four types of biomimetic TPMS heterogeneous structures and applied them to bicycle helmets; among the configurations tested, the Primitive structure exhibited superior performance, attaining the lowest mass (0.2901 kg) and the minimal *HIC* value (79.4). However, we explicitly note that this study does not claim P-TPMS to be the optimal TPMS topology for helmet liner applications. The selection of P-type structure is based on existing evidence from the literature, and a systematic comparative analysis among different TPMS topologies (e.g., Gyroid, Diamond, IWP, Neovius) under identical helmet-impact boundary conditions remains to be conducted in future work. The mathematical representation of the P-TPMS structure is:

(1) f(x,y,z)=cos(2απx)+cos(2βπy)+cos(2γπz)

where *x*, *y*, and *z* denote the Cartesian coordinates in three-dimensional space, and α, β, and γ represent the periodicity parameters along the *x*, *y*, and *z* axes, respectively.

The TPMS, being an ideal surface of negligible thickness, necessitates the creation of a solid structure by topological processing. This work utilizes a shell-based TPMS structure by defining a thickness parameter *t* to ensure that the function values remain within the range of ±*t*. This results in a spatial solid structure with a defined wall thickness (shown in Figure 8b), whose topological representation is the following:

(2) −t≤f(x,y,z)≤t

where *t* is the level-set parameter controlling the offset from the implicit surface. The physical wall thickness (denoted as *C* in the optimization framework) is determined by the offset distance during the solidification process. In this study, the P-TPMS geometry was generated using ANSYS SpaceClaim 2023 R1 (ANSYS, Inc., Canonsburg, PA, USA) to create sheet bodies, followed by STL mesh correction in Geomagic Wrap 2021 (3D Systems, Rock Hill, SC, USA) and solid body generation, with final meshing performed in HyperMesh v11.0. The relative density of the TPMS structure is indirectly influenced by the combination of unit cell size and wall thickness, with larger wall thickness and smaller unit cell size yielding higher relative densities.

**Figure 9:**
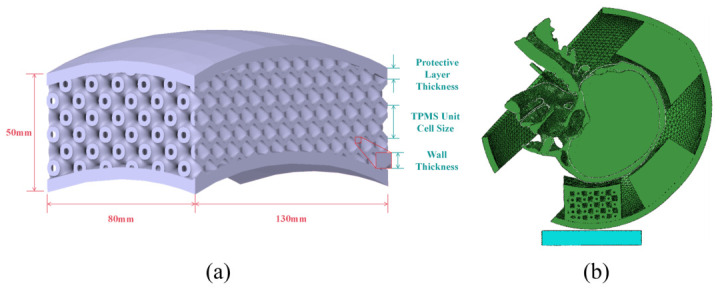
P-TPMS structured helmet: (**a**) geometric model of an individual forehead liner (broadside is not depicted); (**b**) helmet–head coupled finite element model of the complete P-TPMS structure.

This work inserted a P-type array of TPMS into the forehead liner region of the helmet (shown in Figure 9a). The liner measurements were established at 130 mm in length, 80 mm in width, and 50 mm in height, with a geometric configuration tailored to fit the interior curvature of the helmet. A local replacement strategy was implemented instead of a full-liner replacement due to two factors: first, the forehead region is a high-impact area, making targeted reinforcement more cost-effective; second, local application simplifies additive manufacturing and reduces prototype validation costs, thereby facilitating the future design of comprehensive helmet liner.

The forehead liner structure consists of three essential design factors, as illustrated in Figure 9a:

- Outer protective layer thickness (*A*): Acting as a transition layer between the TPMS structure and the original EPS liner, it serves to transfer loads and provide structural support;
- TPMS lattice unit cell length (*B*): These determine the density of the lattice, influencing the structure’s relative density and deformation behavior;
- Wall thickness (*C*): The physical thickness of the TPMS lattice walls in millimeters, which directly relates to structural stiffness and energy absorption capacity.

The interaction of these three parameters greatly affects the impact response characteristics of the liner; systematic optimization will be conducted utilizing the Box–Behnken response surface approach. Figure 9b illustrates the comprehensive P-TPMS helmet–head integrated finite element model.

### 3.2. BBD Response Surface Optimization Analysis

#### 3.2.1. Design of Numerical Experiments

Based on the geometric constraints and manufacturing feasibility of the forehead helmet liner, the outer protective layer thickness (*A*), the TPMS unit cell size (*B*), and the wall thickness (*C*) were selected as the optimization variables. The total height of the cushion is 50 mm, and each parameter must satisfy the following geometric compatibility constraints:

- Outer protective layer thickness and TPMS unit cell size: 2A+B≤50 mm, to ensure spatial feasibility of the structure in the vertical direction;
- Lower limit of TPMS unit cell size: If *B* is too small, it will lead to difficulties in modeling and meshing while also reducing the relative density of the structure.
- Relationship between wall thickness and TPMS unit cell size: 2C≪B, to maintain the topological form of the TPMS units. If *C* is too small, it will significantly weaken the structural stiffness.

Considering the aforementioned limits and integrating them with preliminary screening analysis, each factor was assigned three levels (high, medium, and low), utilizing +1, 0, and −1 coding.

Table 5 displays the factor levels. In contrast to a full factorial design, the BBD markedly decreases the number of tests required while facilitating the estimation of coefficients for linear, quadratic, and interaction terms; it is appropriate for three-factor, multi-level optimization challenges. This study employs *HIC* and *PLA* as response variables, which are the predominant evaluation metrics in the biomechanics of head injury and effectively delineate the risk of brain damage under impact loads. The equation for *HIC* is:

(3) $$\mathrm{HIC}={\left\{\left(t_{2}-t_{1}\right){\left[\frac{1}{t_{2}-t_{1}}\int_{t_{1}}^{t_{2}}a\left(t\right)\;dt\right]}^{2.5}\right\}}_{\max}$$

*a*(*t*) denotes the acceleration of the center of mass of the head (measured in g); $t_{1}$ and $t_{2}$ signify the upper and lower bounds of the time integration, with the condition that $t_{2}-t_{1}\leq 15$ ms.

This work involved the design of 17 numerical simulation runs, comprising five sets of repeated simulations at the center point to assess the model’s goodness of fit. Table 6 presents the simulation schemes and results. The five centre-point simulations yielded slightly different results (runs 8, 10, 11, 14, and 17). These values reflect small numerical variations observed in the simulation workflow and are not assigned statistical significance.

#### 3.2.2. Development of Regression Models and Variance Analysis

Design-Expert v13 (Stat-Ease, Inc., Minneapolis, MN, USA) facilitated multiple regression analysis on the experimental data, creating quadratic polynomial regression models for *HIC* and *PLA*. In the analysis of variance, non-significant variables with *p* > 0.05 were omitted to streamline the model and enhance predictive accuracy; however, the fundamental elements (*A*, *B*, and *C*) were maintained despite their insignificance to uphold the model’s physical interpretation. The precise regression equations for *HIC* and *PLA* are as follows:

(4) $$\mathrm{HIC}=1743.34-94.73A+23.28B-56.56C+9.16AB-82.33AC+165.63BC+73.57B^{2}$$

(5) $$\mathrm{PLA}=217.63-13.71A+3.63B-0.305C+5.75AB-7.59AC+8.28BC+3.02A^{2}+2.22B^{2}$$

The findings of the analysis of variance are presented in Table 7 and Table 8. The *F*-value denotes the ratio of the mean square between groups to the mean square within groups. A highly elevated *F*-value, beyond 1, suggests that the differences among the means are statistically significant. The *p*-value is the probability, under the null hypothesis, of obtaining a test statistic at least as extreme as the observed one, used to evaluate the statistical significance of the model terms. A smaller *p*-value signifies a more significant coefficient. *p* < 0.01 denotes high significance, while *p* < 0.05 indicates significance.

Table 7 illustrates that the *F*-value for the *HIC* model is 77.95, with *p* < 0.0001, signifying high significance; the *p*-value for the non-linearity term is 0.6887, which exceeds 0.05, showing that the non-linearity term is not significant and that the model demonstrates a satisfactory fit. Among the linear terms, factors *A* and *C* exhibit *p* < 0.0001, signifying a highly significant impact on *HIC*; factor B shows *p* = 0.0196, suggesting a substantial effect. The *p*-values for the interaction terms *AB*, *AC*, and *BC* are all below 0.0001, signifying that the two-way interactions among the three factors exert a highly significant influence on *HIC*. The *p*-value for the quadratic term *B*^2^ is 0.0001, signifying that unit cell size exerts a substantial quadratic influence on *HIC*.

Table 8 illustrates that the *F*-value for the *PLA* model is 32.41, with *p* < 0.0001, signifying high significance; conversely, the *p*-value for the residual term is 0.6931, exceeding 0.05, showing insignificance and confirming a good model fit. For factor *A*, *p* < 0.0001, signifying a highly significant effect on *PLA*; for factor *B*, *p* = 0.0089, denoting a substantial effect; and for factor *C*, *p* = 0.7799, indicating no significant effect. The *p*-values for the interaction terms *AB*, *AC*, and *BC* are all below 0.01, signifying that the interactions among factors significantly influence *PLA*.

The model fit was assessed using statistical metrics including $R^{2}$, adjusted $R^{2}$, projected $R^{2}$, and coefficient of variation (C.V.%), as presented in Table 9. The $R^{2}$ for the *HIC* model was 0.9838, and the $R^{2}$ for the *PLA* model was 0.9701. While these values indicate good fit within the training data, it should be noted that the model was developed based on only 17 simulation points within a limited design space, and the predictive accuracy for extrapolation beyond this space requires cautious interpretation. The discrepancies in adjusted $R^{2}$ and predicted $R^{2}$ for both models were small, suggesting that the models exhibit reasonable predictive efficacy within the evaluated design space. The coefficients of variation were 1.31% and 1.36%, respectively, and the signal-to-noise ratios were 31.74 and 21.32, both exceeding the threshold of 4.

**Figure 10:**
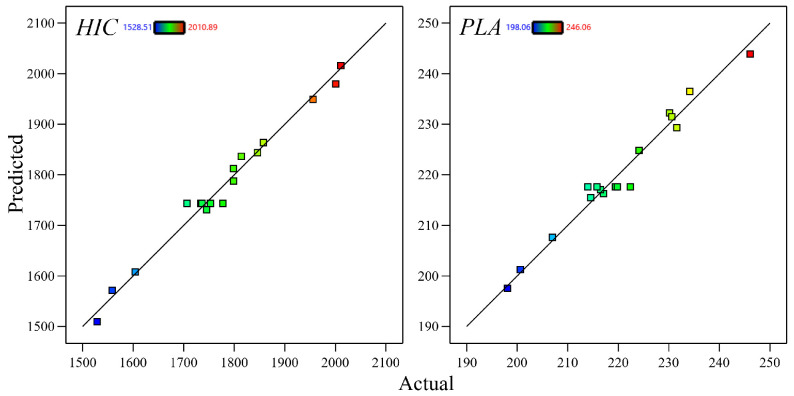
Plots of the actual and predicted values for *HIC* and *PLA*.

**Figure 11:**
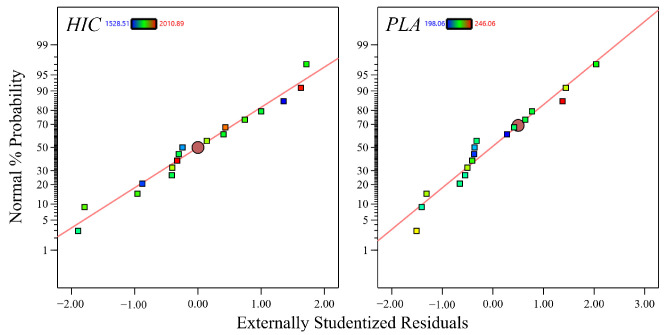
Plots of the normal probability and externally studentized residuals for *HIC* and *PLA*.

Figure 10 presents the scatter plots of actual versus predicted values for *HIC* and *PLA*, with data points aligned along the 45° reference line indicating good agreement. The residual analysis (Figure 11) confirms that residuals follow a normal distribution without systematic bias, validating the regression model assumptions. Collectively, Figure 10 and Figure 11 provide a comparative assessment of actual versus predicted values: the scatter plots demonstrate close agreement between simulated and predicted responses, while the residual analysis indicates no discernible systematic prediction bias.

**Figure 12:**
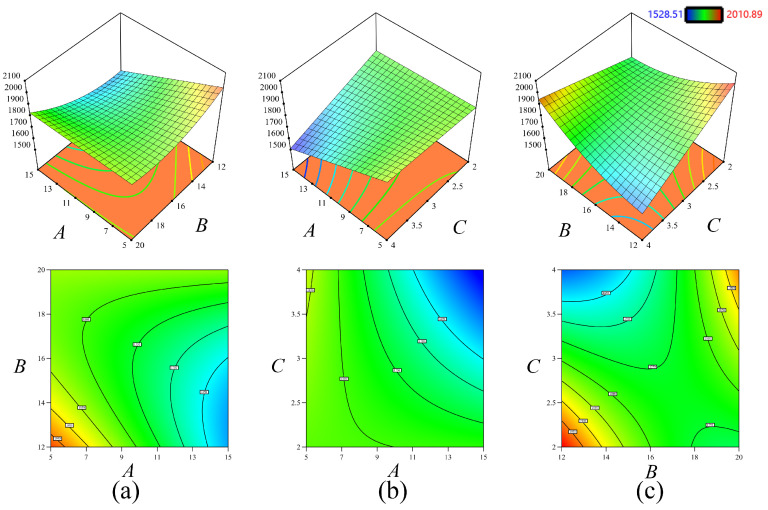
Response surface graphs and contour plots of *HIC*: the effect of (**a**) outer protective layer thickness; (**b**) TPMS unit cell size; (**c**) wall thickness.

**Figure 13:**
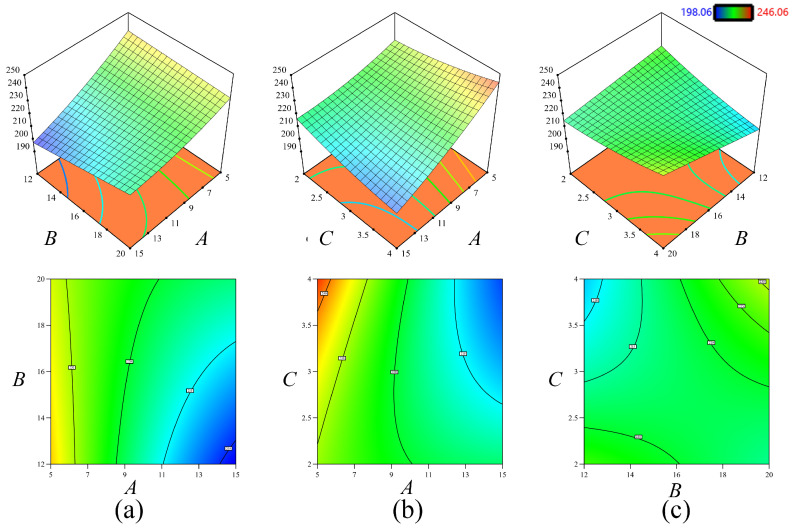
Response surface graphs and contour plots of *PLA*: effect of (**a**) outer protective layer thickness; (**b**) TPMS unit cell size; (**c**) wall thickness.

**Figure 14:**
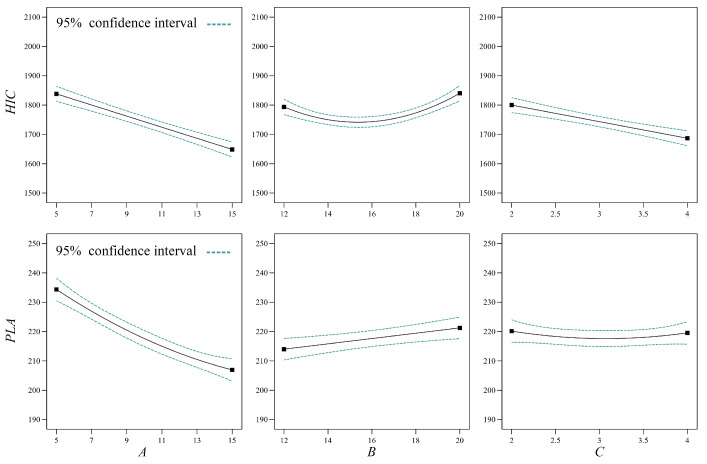
Main effects plot for *HIC* and *PLA* effect of outer protective layer thickness (*A*), TPMS unit cell size (*B*), and wall thickness (*C*).

Figure 12, Figure 13 and Figure 14 present a systematic qualitative analysis of the influence of outer protective layer thickness (*A*), TPMS unit cell size (*B*), and wall thickness (*C*) on *HIC* and *PLA*, utilizing response surface plots, contour plots, and main effects plots.

The response surface plot indicates that augmenting *A* and *C* substantially diminishes *HIC*, whereas elevating *B* results in an increase in *HIC*. The main effects plot corroborates the predominant adverse effect of *A* on *HIC*, with B displaying a quadratic effect (featuring an optimal value) and *C* also exerting a negative impact. The interactions of *A* and *C*, *B* and *C*, and *A* and *B* are all significant: augmenting *A* alleviates the detrimental effect of increasing *B* on *HIC*; the enhancement from raising *C* is more pronounced when *A* is minimal, and *B* and *C* must be synchronized to manage impact loads. The response surface plot indicates that a rise in *A* markedly decreases *PLA*, an increase in *B* marginally elevates *PLA*, and *C* exerts no significant influence; the main effects plot corroborates the predominant negative impact of *A*, the positive influence of *B*, and the insignificance of *C*. The interactions among *A* and *C*, *B* and *C*, and *A* and *B* are all substantial. An increase in *A* can mitigate the acceleration increase induced by *B*; wall thickness necessitates a thin outer protective layer thickness to diminish *PLA*, whereas large element dimensions demand thick-walled support.

The trends of the primary effects of each factor align with the patterns of interaction. *A* is the principal determinant in modulating *HIC* and *PLA*, whereas the interplay between *B* and *C* enhances the response mechanism of impact performance.

#### 3.2.3. Multi-Objective Optimization and Validation

Utilizing Design-Expert v13 software, a multi-objective optimization function was established to concurrently minimize *HIC* and *PLA* with equal weighting. The system produced 100 sets of Pareto front solutions. Table 10 displays 20 representative results selected from these, including the optimal solution (desirability D = 1.000), a suboptimal solution, and boundary solutions with desirability D < 1, to thoroughly illustrate the distribution characteristics of the optimization space.

**Figure 15:**
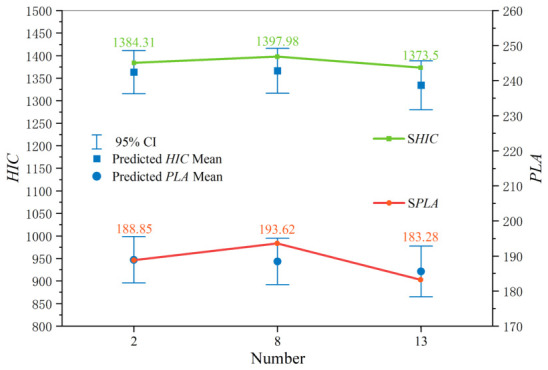
Validation of numerical simulation outcomes.

Model validation is a crucial process for confirming the dependability of regression model extrapolation. The parameter combinations associated with the ideal and sub-optimal outcomes for the combined *HIC* and *PLA* (numbers 2, 8, and 13) were chosen for finite element validation. Following the rounding of each parameter to two decimal places, the predicted values from the regression model were derived using the point prediction function in Design-Expert v13 and subsequently imported into the finite element model for numerical simulation, resulting in the simulated values. Figure 15 illustrates that the simulated *HIC* values (S*HIC*) and predicted *HIC* values (P*HIC*), along with the simulated *PLA* values (S*PLA*) and predicted *PLA* values (P*PLA*), are included within the 95% confidence interval. The validation outcomes are detailed in Table 11.

The developed regression model exhibits significant predictive accuracy and reliability for *HIC* and *PLA*, enabling swift optimization of structural parameters and performance forecasting for TPMS forehead helmet liner structures. It should be noted that the parameter values in Table 10 are presented with three decimal places as the direct output of the Design-Expert v13 optimization algorithm. For the subsequent finite element validation, these values were rounded to two decimal places using the point prediction function in Design-Expert v13, as shown in Table 11. However, it should be acknowledged that these validation points were selected from the Pareto front solutions and are not completely independent from the model fitting process. Additional validation using independent design points would strengthen the model reliability assessment.

## 4. Analysis of the Protective Efficacy of TPMS Helmets

This section selects three optimized parameter combinations (numbers 2, 8, and 13) for systematic comparison with the original EPS liner, based on the results of the BBD optimization validation, to evaluate the impact protection performance of the TPMS forehead liner from the dual perspectives of head kinematic response and head biomechanical response. The assessment measures comprise *HIC*, *PLA*, *SFC*, *ICP*, *MPS*, and AIS2+.

### 4.1. Kinematic Response of the Head

**Figure 16:**
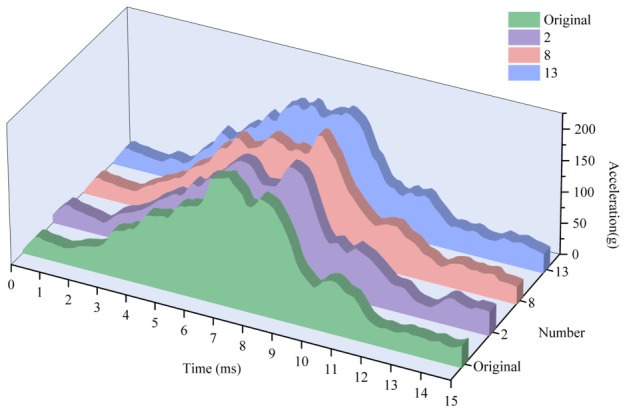
Comparison chart of head center of mass acceleration.

The head kinematic response is the principal criterion for assessing helmet protective efficacy. Figure 16 illustrates the time-history curves of the head center-of-mass acceleration for the original helmet and the three optimized designs, while Table 12 provides a comparison of essential kinematic characteristics.

To assess whether the performance improvement is attributable to structural advantages rather than increased mass, a mass comparison was conducted between the original EPS forehead liner and the optimized TPMS liner. The original EPS forehead liner has a mass of 41.6 g, while the optimal TPMS liner (Scheme 13) has a mass of 39.4 g, representing a mass reduction of approximately 5.3%. This indicates that the superior protective performance of the TPMS liner is achieved with a lighter structure, demonstrating improved mass efficiency rather than simply increasing material volume.

The *HIC* values for all three optimized designs were markedly lower than that of the original helmet (1634.62), with reductions between 14.43% and 15.98%. Scheme 13 exhibited the lowest *HIC* value (1373.50), reflecting a 15.98% decrease relative to the original helmet, signifying that this parameter combination is the most efficacious in mitigating the risk of cumulative head injury. The notable decrease in *HIC* is ascribed to the distinctive progressive energy absorption mechanism of the P-TPMS structure: under impact loads, the TPMS lattice persistently dissipates energy via layer-by-layer buckling deformation, thereby averting the abrupt increase in acceleration resulting from the swift compaction of the EPS liner.

The *PLA* values for all three optimized systems were inferior to that of the original helmet (213.63), with reductions between 9.36% and 14.21%. The peak time was postponed from 7.05 ms for the original helmet to 8.25–8.40 ms, resulting in a delay of 1.20–1.35 ms. The postponement in the *PLA* timing signifies that the TPMS configuration successfully extends the impact duration, facilitating gradual energy absorption over an extended timeframe, which corresponds with the core protective principle of “lengthening the impact duration to diminish peak load”. Scheme 13 demonstrated the lowest *PLA* (183.28 g) and a deferred peak time of 8.40 ms, attaining the ideal equilibrium between energy absorption efficacy and impact attenuation performance.

Evaluation of cranial fracture susceptibility. The *SFC* was established to estimate the risk of skull fracture. According to the research findings of Vorst et al. [49] about the correlation between skull fracture risk and fracture-related markers, the method for calculating the *SFC* is as follows:

(6) $$\mathrm{SFC}=\frac{\int_{t_{1}}^{t_{2}}a_{t}\;dt}{t_{2}-t_{1}}$$

Here, $a_{t}$ signifies the acceleration of the head’s center of mass over time (in g), while $t_{1}$ and $t_{2}$ indicate the upper and lower bounds of the time integration, with $t_{2}-t_{1}=15$ ms.

**Figure 17:**
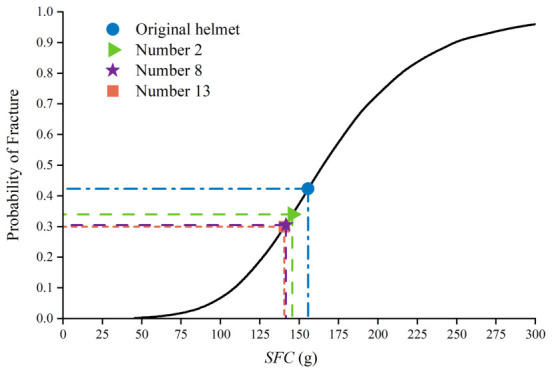
Prediction results of skull fracture probability under different configurations.

Table 13 and Figure 17 illustrate that the *SFC* of the original helmet is 155.74, which correlates to an approximate skull fracture risk of 42%. The *SFC* values for the three improved methods were decreased to 145.73 (34%), 141.69 (30.5%), and 140.69 (30%), respectively. Scheme 13 exhibited the most significant reduction in skull fracture risk, with a fall of 12 percentage points, demonstrating that the P-type cushioning system with TPMS for the forehead can effectively mitigate the risk of skull fracture.

In conclusion, all three optimization strategies exhibited outstanding performance across the three dynamic metrics: *HIC*, *PLA*, and *SFC*. Scheme 13 demonstrated the most superior performance, decreasing *HIC* by 15.98% and *PLA* by 14.21% while reducing the probability of a skull fracture from 42% to 30%, thereby significantly improving the helmet’s impact protection efficacy.

### 4.2. Biomechanical Response of the Head

The biomechanical response of the head is essential for elucidating the risk of deep cranial injury. This section analyzes the response from the viewpoints of *ICP* and *MPS* and further assesses the risk of AIS2+ based on *MPS*.

**Figure 18:**
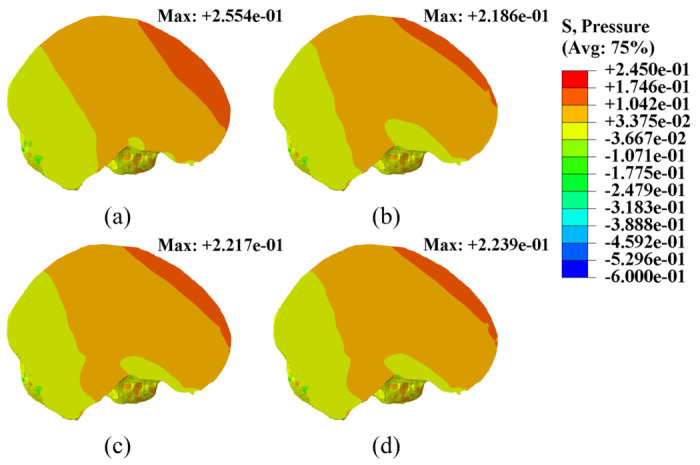
*ICP* contour maps comparison: (**a**) original; (**b**) number 2; (**c**) number 8; (**d**) number 13.

*ICP* analysis. Figure 18 illustrates the pressure distribution contour map of the *ICP* subjected to a flat-anvil impact scenario. Figure 18a illustrates that the peak *ICP* of the original helmet is 255.4 kPa, with high-pressure zones (red regions) centered at the anterior region of the brain, arranged in a narrow, elongated strip exhibiting a pronounced pressure gradient. Tse et al. [50] claim that *ICP* over 250 kPa signifies a danger of cerebral injury, indicating that the original helmet is at the injury threshold under these circumstances.

All three optimization approaches significantly lowered the *ICP* (Figure 18b–d). The maximum intracranial pressures for designs 2, 8, and 13 were 218.6 kPa, 221.7 kPa, and 223.9 kPa, respectively, indicating decreases of 14.4%, 13.2%, and 12.2%, all remaining below the damage threshold of 250 kPa. The optimized helmets exhibited a notable reduction in the dimensions of high-pressure zones; the area of the red high-pressure regions diminished, while the yellow low-pressure regions expanded towards the front, signifying that the TPMS structure effectively enhanced the spatial uniformity of pressure distribution. Sample 2 displayed the most significant decrease in *ICP* and the most notable shrinking of the high-pressure zone, indicating superior pressure cushioning ability.

**Figure 19:**
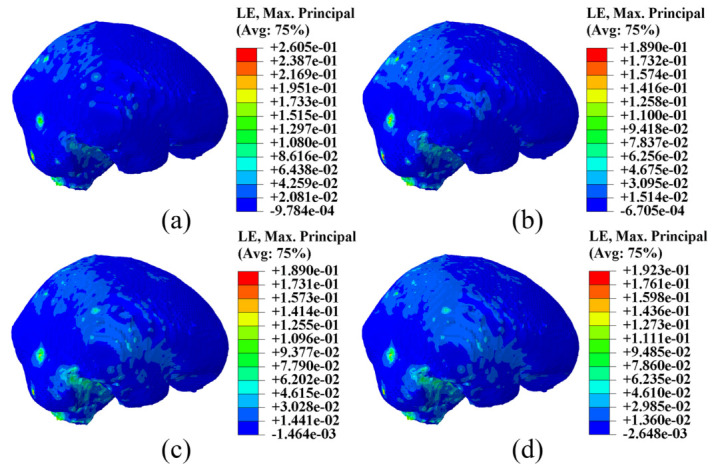
*MPS* contour maps comparison: (**a**) original; (**b**) number 2; (**c**) number 8; (**d**) number 13.

Analysis of *MPS*. Figure 19 illustrates the cranial strain cloud maps across several parameter settings. Figure 19a illustrates that the peak *MPS* of the original helmet is 0.261, with the area of maximal strain situated at the interface of white and grey matter at the posterior region of the brain. Research conducted by Galbraith et al. [51] and Shreiber et al. [52] indicates that an *MPS* above 0.25 may result in significant structural damage to the central nervous system, suggesting that the original helmet presents a considerable danger of TBI.

Each of the three sets of improved settings markedly diminished the *MPS* (Figure 19b–d). The maximum *MPS* values for sets 2, 8, and 13 were 0.189, 0.189, and 0.192, respectively, indicating decreases of 27.6%, 27.6%, and 26.4%, all of which fall below the functional injury threshold of 0.20. The improved helmet exhibited a large reduction in the area of high strain, concomitantly leading to a substantial increase in the proportion of the blue low-strain region, thus achieving a more uniform strain distribution. This suggests that the P-TPMS liner for the forehead can efficiently diminish brain tissue deformation and substantially lower the risk of TBI.

**Figure 20:**
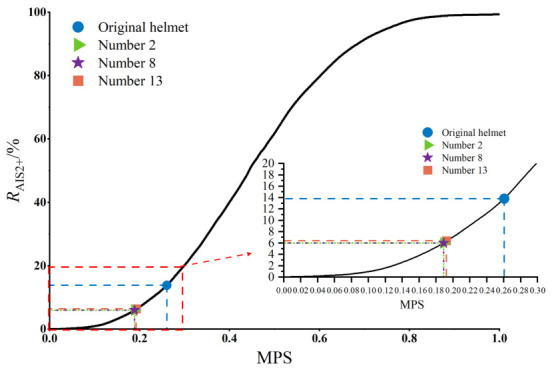
Comparison of AIS2+ under different configurations.

To easily quantify the risk of TBI, the likelihood of AIS2+ (*R*_AIS2+_%) for each configuration was computed based on the *MPS* values obtained from the simulation, utilizing the correlation curve between *MPS* and AIS2+ probability [53]. Figure 20 illustrates the findings: the *R*_AIS2+_% for the original helmet was roughly 14%, signifying a substantial risk of mTBI; the *R*_AIS2+_% for the three improved designs decreased to 6.0%, 6.0%, and 6.4%, respectively, reflecting a reduction of 7.6 to 8.0 percentage points. Among them, schemes 2 and 8 displayed the lowest *R*_AIS2+_% (6.0%), signifying a reduction of roughly 57% relative to the original helmet, demonstrating that the P-TPMS forehead liner exhibits superior efficacy in safeguarding against brain tissue injury.

**Figure 21:**
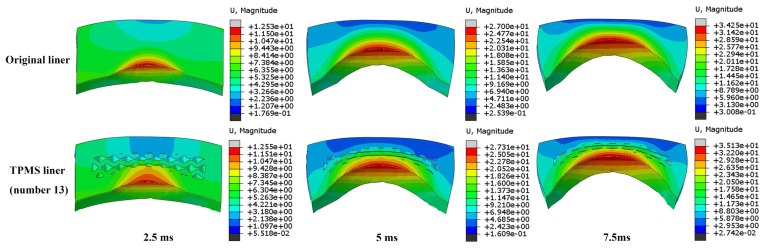
Displacement evolution comparison: original EPS liner and P-TPMS liner (Scheme 13) at 2.5 ms, 5 ms, and 7.5 ms.

To provide direct evidence of the deformation mechanism underlying the improved protective performance, Figure 21 compares the displacement evolution of the original EPS liner and the optimal P-TPMS liner (Scheme 13) at three time points during impact. For the original EPS liner, deformation is highly localized beneath the impact region, with the high-displacement zone rapidly expanding from the center and forming a concentrated collapse pattern. By 7.5 ms, the central region approaches full compression, exhibiting a global collapse mode typical of foam materials under high-energy impact. In contrast, the P-TPMS liner exhibits a sequential buckling process characterized by the progressive collapse of multiple P-TPMS cells. At 2.5 ms, localized buckling is already observable in the P-TPMS layer, indicating early engagement of the energy-absorbing structure. As the impact progresses, the deformation gradually spreads along the liner rather than remaining confined to the impact center, demonstrating a distributed deformation mechanism. This progressive layer-by-layer buckling behavior promotes more effective energy dissipation and delays localized densification, thereby explaining the reduced peak acceleration and extended impact duration observed in the kinematic response analysis.

In conclusion, the three sets of optimized parameters exhibited outstanding performance across the three biomechanical indicators—*ICP*, *MPS*, and AIS2+. *ICP* diminished by 12.2% to 14.4%, all remaining below the injury threshold of 250 kPa; *MPS* reduced by 26.4% to 27.6%, all below the functional injury threshold of 0.20; and *R*_AIS2+_% decreased from 14% to 6.0–6.4%, indicating a reduction exceeding 50%. Set 13 exhibited the most superior performance, significantly diminishing the risk of TBI.

## 5. Discussion

The locally reinforced P-TPMS forehead helmet liner presented in this work exhibits considerable benefits regarding impact protection performance. The enhancement in its protective efficacy can be elucidated through its structural topological features and energy dissipation mechanisms.

From the standpoint of structural topological attributes, the P-TPMS exhibits a seamless, continuous curved surface geometry, hence mitigating the stress concentration problems typically associated with conventional porous materials. During impact stress, the TPMS lattice experiences gradual, layer-by-layer buckling deformation, in contrast to the abrupt failure seen in EPS foam. This progressive collapse behavior is directly evidenced by the displacement evolution analysis (Figure 21), which shows that the TPMS liner exhibits sequential buckling with deformation spreading along the structure, whereas the original EPS liner displays localized concentration and rapid collapse. The disparity in deformation behavior directly influences the shape of the acceleration response curve: the TPMS structure produces a more gradual increase in acceleration and a markedly postponed peak, thus effectively diminishing the *HIC* value. The described mechanism aligns with the tensile-buckling-dominated deformation mode of P-type lattices identified by Maskery et al. [14], elucidating the fundamental cause of its superior energy absorption efficiency relative to bending-dominated structures (such as Gyroid and Diamond).

The elevated pore connectivity of the TPMS structure facilitates homogeneous stress distribution during compression, enhancing energy dissipation mechanisms. Traditional EPS liners are susceptible to localized densification during high-velocity impacts, leading to a “bottom-out” effect that disrupts energy absorption. Conversely, the TPMS structure can perpetually dissipate impact energy via the gradual buckling and collapse of lattice walls, thus postponing the densification process. This attribute markedly postpones the *PLA* peak period, validating the established premise in impact protection that “prolonging the impact length diminishes peak load”.

This study presents a localized heterogeneous reinforcement design paradigm specifically for the forehead region, in contrast to the monolithic TPMS liner replacement technique employed by Liu et al. [16]. This strategy is predicated on two factors: firstly, since the forehead is the most commonly affected region of the helmet (constituting approximately 19% of all impacts), focused reinforcement can provide optimal protective advantages at minimal mass expense, thus enhancing the cost–effectiveness ratio; secondly, localized replacement markedly diminishes the complexity and material expenses associated with additive manufacturing, thereby streamlining engineering execution. Notably, the optimal TPMS design achieves a 5.3% mass reduction compared to the original EPS liner while simultaneously improving protective performance across all evaluated metrics, demonstrating that the improvement is attributable to structural efficiency rather than increased material volume. This study’s results confirm the viability of this method, showing that local optimization of the forehead liner has led to a synergistic decrease in various damage measures, including *HIC*, *PLA*, *ICP*, *MPS*, and AIS2+.

Several limitations of this study should be acknowledged. First, the BBD response surface optimization is based entirely on deterministic numerical simulations without physical experimental random error; the pure error term in the ANOVA tables solely reflects the solver’s inherent computational variability and should not be interpreted as an estimate of experimental reproducibility. Second, the selection of the P-TPMS topology was based on existing literature evidence rather than a systematic comparative analysis under identical helmet-impact boundary conditions; therefore, it cannot be concluded that P-TPMS is the optimal TPMS architecture for helmet liner applications. Third, the TPMS structure was simulated using the same EPS crushable foam material model as the original liner. While this enables isolation of geometric effects, the actual constitutive response of additively manufactured polymers may differ in strain-rate sensitivity, failure modes, and scale effects. Fourth, the model validation was conducted only for the helmet–head coupled model with the original EPS liner (peak acceleration error 8.4%, *HIC* error 11.6%), and the predictive accuracy for the TPMS liner remains to be verified through physical impact tests. Furthermore, this validation error is comparable in magnitude to the performance differences between some optimized designs (e.g., the *PLA* difference between cases 7 and 8 in Table 6 is approximately 6.2%). This implies that while relative optimization trends are reliable, the absolute predictive accuracy for individual designs should be interpreted with caution, and the precise magnitude of improvement requires physical validation. Fifth, the present study is confined to frontal flat-anvil impact scenarios; findings from this single configuration are insufficient to evaluate the overall protective efficacy under diverse impact locations and oblique loading conditions. Sixth, this study focuses on geometric design and simulation, omitting additive manufacturing considerations such as process-induced defects, geometric tolerances, anisotropic mechanical behavior from layer-by-layer deposition, and stress concentrations at the TPMS–EPS interface, all of which may influence the actual energy absorption performance. Seventh, a comprehensive multi-criteria evaluation including manufacturing cost, production time, and user comfort factors has not been conducted; the additive manufacturing cost of TPMS liners is considerably higher than that of EPS compression molding, which may limit commercial viability to premium helmet products. Eighth, the multi-objective optimization employed equal weighting for *HIC* and *PLA* without sensitivity analysis on the weighting coefficients, and the optimal parameter combination lies close to the upper boundary of the design space (outer protective layer thickness of 14.95 mm versus the 15 mm upper limit), which warrants caution regarding optimization stability and robustness to weighting variations.

Future research may focus on the following domains: (1) conducting systematic comparative analyses among different TPMS topologies (e.g., Gyroid, Diamond, IWP, Neovius) under identical helmet-impact boundary conditions to identify the most suitable architecture for helmet liner applications; (2) developing and validating material models for additively manufactured polymers (e.g., TPU, PA12, Polylactic acid) through experimental characterization at various strain rates, and examining the effects of manufacturing precision on energy absorption; (3) performing impact assessments at multiple locations and angles, with particular emphasis on oblique impacts, to evaluate rotational kinematic responses and rotational injury criteria alongside translational metrics; (4) fabricating physical prototypes of the optimal TPMS liner design and conducting drop-tower impact tests following ECE 22.06 standard procedures to validate the computational predictions; and (5) conducting sensitivity analysis on the weighting coefficients in the multi-objective optimization and developing a multi-objective trade-off model that incorporates manufacturing costs, protective efficacy, and weight-saving advantages to evaluate commercial feasibility.

## 6. Conclusions

This study addresses the problems of “bottom-out” and inadequate energy absorption in conventional EPS helmet liners during high-energy impacts by proposing a locally reinforced forehead energy-absorbing liner utilizing a P-TPMS structure and systematically assessing its impact protection efficacy. The primary conclusions are as follows:

1. A helmet–head coupled model was developed via inverse modeling, based on a validated high-fidelity finite element model of the head. Simulations of drop tests performed in compliance with the ECE 22.06 standard indicated that the results were consistent with Hybrid III dummy test data about acceleration response trends, confirming the accuracy and reliability of the coupling model.
2. By choosing the outer protective layer thickness, the TPMS unit cell size, and the wall thickness as design variables, a quadratic regression prediction model for *HIC* and *PLA* was developed utilizing the BBD response surface method, achieving coefficients of determination $R^{2}$ of 0.9838 and 0.9701, respectively, with prediction errors remaining below 3%. The ideal parameter combination identified through multi-objective optimization was an outer protective layer thickness of 14.95 mm, a TPMS unit cell size of 12.23 mm, and a wall thickness of 3.93 mm.
3. The optimal P-TPMS liner, in comparison to the original EPS liner, resulted in a 15.98% reduction in *HIC* and a 14.21% reduction in *PLA*. Additionally, the probability of skull fracture decreased from 42% to 30%. In terms of biomechanical efficiency, the *ICP* was reduced by 12.2% from 255.4 kPa to 223.9 kPa, transitioning from above the 250 kPa cerebral injury threshold to below it, thereby eliminating the risk of intracranial pressure-induced brain injury under the evaluated impact condition. The *MPS* was reduced by 26.4% from 0.261 to 0.192, crossing from above the 0.20 functional injury threshold to below it, indicating that the P-TPMS liner effectively prevents structural damage to the central nervous system. Furthermore, AIS2+ decreased from 14% to 6.0%, reflecting a substantial reduction in the risk of mild traumatic brain injury.
4. The P-TPMS structure consistently dissipates impact energy via layer-by-layer buckling deformation, effectively extending the impact duration, mitigating the abrupt acceleration surge from the rapid compaction of the EPS liner, and markedly improving the helmet’s impact protection efficacy.

This study demonstrates the application of P-TPMS structures in a localized helmet liner reinforcement design. The selection of P-type topology is based on existing literature evidence, and this study does not claim that P-TPMS is the optimal TPMS architecture for helmet liner applications. The results provide a reference for the development of spatially heterogeneous liners in protective equipment, although further validation through physical experiments, evaluation under diverse impact conditions, and comparative analysis with alternative TPMS topologies are warranted. It should be noted that the TPMS liner was modelled using the same EPS crushable foam material model as the original liner to isolate geometric effects; the reported results therefore represent the structural contribution of the P-TPMS architecture rather than predictions for a specific additively manufactured polymer such as TPU, PA12, or Polylactic acid.

## Figures and Tables

**Table 1:** Head Model Material Parameters [20].

	Young’s Modulus *E* (MPa) $G\left(t\right)=G_{\infty }+\left(G_{0}-G_{\infty }\right)e^{-\beta t}$	Poisson’s Ratio ν	Density ρ (g/cm^3^)	Reference
Brainstem	$G_{0}=0.528$, $G_{\infty }=0.168$, β=35 s^−1^	0.48	1.14	[20,25,26,27,28,29,30,31]
Cerebral peduncle	$G_{0}=0.0225$, $G_{\infty }=0.0045$, β=80 s^−1^	0.4996	1.06	[32]
Cerebrum	$G_{0}=0.528$, $G_{\infty }=0.168$, β=35 s^−1^	0.48	1.14	[20,25,26,27,28,29,30,31]
Cerebellum	$G_{0}=0.528$, $G_{\infty }=0.168$, β=35 s^−1^	0.48	1.14	[20,25,26,27,28,29,30,31]
Cerebrospinal fluid	1.314	0.4999	1.04	[31]
Gray matter	$G_{0}=0.528$, $G_{\infty }=0.168$, β=35 s^−1^	0.4996	1.04	[20,32]
Lateral cartilage	30	0.45	1.50	[33]
Septal cartilage	9	0.32	1.50	[34]
Bone	8000	0.22	4.74	[20]
Soft tissues	16.67	0.46	1.04	[20,35]
Tooth	2070	0.3	2.25	[36,37]
Ventricles	1.314	0.4999	1.04	[31]
White matter	$G_{0}=0.041$, $G_{\infty }=0.0078$, β=700 s^−1^	0.4996	1.04	[32]

**Table 2:** Material Parameters for Helmet Components and TPMS Liner [46].

	Young’s Modulus *E* (MPa)	Poisson’s Ratio ν	Density ρ (g/cm^3^)	Yielding Strength (MPa)
Shell	*E* = 2 × 10^3^	0.37	1.15	34.3
Energy-absorbing liner	*E* = 20, $\sigma_{Y}$ = 0.6, k=1.933, $k_{t}=0.1$	0.01	0.08	-
TPMS forehead liner	*E* = 20, $\sigma_{Y}$ = 0.6, k=1.933, $k_{t}=0.1$	0.01	0.08	-
Straps	*E* = 2 × 10^3^	0.42	1.10	-
Anvil	*E* = 2.1 × 10^5^	0.3	7.80	-

**Table 3:** Finite element modelling details for helmet–head coupled model.

Component	Element Type	Mesh Size	Contact Definition
Shell	Hexahedral (C3D8R)	5 mm	Shell-anvil: surface-to-surface
EPS liner	Tetrahedral (C3D4)	3 mm	Liner-shell: tied constraint
TPMS liner	Tetrahedral (C3D4)	3 mm	TPMS-shell: tied constraint
Straps	Hexahedral (C3D8R)	5 mm	Straps-shell: tied constraint
Anvil	Hexahedral (C3D8R)	Rigid body	Anvil-ground: fixed

**Table 4:** Mesh convergence analysis results for helmet–head coupled model with original EPS liner.

Mesh Density	Element Size	*HIC*	*PLA* (g)	Computational Time
Coarse	5 mm	1742.5	228.7	2.0 h
Medium	3 mm	1634.62	213.63	6.2 h
Fine	2 mm	1612.8	209.4	18 h
Variation (Medium vs. Fine)		1.4%	2.0%	–

**Table 5:** BBD response surface test factor levels.

Coded	*A*: Outer Protective Layer Thickness	*B*: TPMS Unit Cell Size	*C*: Wall Thickness
+1	15 mm	20 mm	4 mm
0	10 mm	16 mm	3 mm
−1	5 mm	12 mm	2 mm

**Table 6:** BBD response surface numerical simulation scheme and results.

Number	*A*	*B*	*C*	*HIC*	*PLA* (g)
1	15	16	2	1798.54	216.48
2	10	20	4	1955.76	230.57
3	5	16	2	1798.37	231.57
4	10	12	2	2010.89	224.09
5	5	20	3	1845.89	230.13
6	5	16	4	1857.66	246.06
7	15	16	4	1528.51	200.61
8	10	16	3	1706.73	219.80
9	15	20	3	1813.78	217.05
10	10	16	3	1777.45	215.77
11	10	16	3	1752.68	222.36
12	5	12	3	2000.81	234.13
13	10	12	4	1558.77	206.99
14	10	16	3	1736.3	213.99
15	10	20	2	1745.38	214.53
16	15	12	3	1604.07	198.06
17	10	16	3	1733.86	219.45

**Table 7:** ANOVA results for the coefficient of quadratic model for *HIC*.

Source	Sum of Squares	Degrees of Freedom	Mean Square	*F*-Value	*p*-Value
Model	2.947 × 10^5^	7	42,103.18	77.95	<0.0001
*A*	71,788.29	1	71,788.29	132.91	<0.0001
*B*	4337.06	1	4337.06	8.03	0.0196
*C*	25,592.27	1	25,592.27	47.38	<0.0001
*AB*	33,238.76	1	33,238.76	61.54	<0.0001
*AC*	27,112.92	1	27,112.92	50.20	<0.0001
*BC*	1.097 × 10^5^	1	1.097 × 10^5^	203.16	<0.0001
*B* ^2^	22,926.40	1	22,926.40	42.45	0.0001
Residual	4861.00	9	540.11	-	-
Lack of Fit	2149.29	5	429.86	0.6341	0.6887
Pure Error	2711.71	4	677.93	-	-
Cor Total	2.996 × 10^5^	16	-	-	-

The BBD is based entirely on deterministic numerical simulations. The “Pure Error” term represents the inherent numerical discretization of the finite element solver rather than physical experimental error.

**Table 8:** ANOVA results for the coefficient of the quadratic model for *PLA*.

Source	Sum of Squares	Degrees of Freedom	Mean Square	*F*-Value	*p*-Value
Model	2309.82	8	288.73	32.41	<0.0001
*A*	1503.99	1	1503.99	168.81	<0.0001
*B*	105.20	1	105.20	11.81	0.0089
*C*	0.7442	1	0.7442	0.0835	0.7799
*AB*	132.14	1	132.14	14.83	0.0049
*AC*	230.43	1	230.43	25.86	0.0009
*BC*	274.56	1	274.56	30.82	0.0005
*A* ^2^	38.54	1	38.54	4.33	0.0711
*C* ^2^	20.88	1	20.88	2.34	0.1644
Residual	71.27	8	8.91	-	-
Lack of Fit	26.25	4	6.56	0.5828	0.6931
Pure Error	45.03	4	11.26	-	-
Cor Total	2381.09	16	-	-	-

The BBD is based entirely on deterministic numerical simulations. The “Pure Error” term represents the inherent numerical discretization of the finite element solver rather than physical experimental error.

**Table 9:** Statistical parameters from the ANOVA for the models.

Response	Std. Dev.	Mean	C.V. %	$R^{2}$	Adjusted $R^{2}$	Predicted $R^{2}$	Adequate Precision	PRESS
*HIC*	23.24	1777.97	1.31	0.9838	0.9712	0.9391	31.7446	18253
*PLA*	2.98	220.10	1.36	0.9701	0.9401	0.8703	21.3209	308.71

Std. Dev: standard deviation, C.V.: coefficient of variance, PRESS: predicted residual error sum of squares.

**Table 10:** Parameters list of Design-Expert v13 prediction.

Number	Outer Protective Layer Thickness	TPMS Unit Cell Size	Wall Thickness	*HIC*	*PLA* (g)	Desirability
1	14.384	13.657	3.573	1495.608	196.81	1.000
2	14.949	12.975	3.915	1364.774	188.944	1.000
3	13.061	13.29	3.935	1460.520	197.589	1.000
4	14.98	13.33	3.802	1405.797	191.413	1.000
5	14.415	12.135	3.346	1528.018	194.540	1.000
6	13.538	13.513	3.833	1468.820	197.183	1.000
7	14.843	13.422	3.381	1517.533	196.624	1.000
8	14.406	12.381	3.934	1365.888	188.438	1.000
9	12.290	12.485	3.972	1466.638	197.835	1.000
10	14.134	13.182	3.756	1449.494	194.334	1.000
11	13.475	13.705	3.853	1473.844	197.988	1.000
12	14.742	13.309	3.658	1451.02	193.592	1.000
13	14.951	12.23	3.928	1334.721	185.635	1.000
14	13.785	13.416	3.78	1466.449	196.281	1.000
15	14.197	12.911	3.525	1497.409	195.465	1.000
16	12.520	12.507	3.919	1468.150	197.221	1.000
17	14.990	12.061	3.280	1523.073	193.415	1.000
18	5.267	20.000	2.000	1656.583	218.559	0.649
19	5.144	20.000	2.000	1654.654	218.710	0.649
20	5.319	19.999	2.000	1657.429	218.499	0.649

**Table 11:** Simulation results and prediction errors.

Number	P*HIC*	S*HIC*	*HIC* Error	P*PLA* (g)	S*PLA* (g)	*PLA* Error
2	1363.72	1384.31	0.0151	188.915	188.85	−0.04%
8	1366.69	1397.98	0.0229	188.456	193.62	2.74%
13	1334.34	1373.5	0.0293	185.624	183.28	−1.26%

**Table 12:** Head kinematic parameter statistics.

Number	*HIC*	*PLA* (g)	Time Point of *PLA* (ms)
Original	1634.62	213.63	7.05
2	1384.31	188.85	8.25
8	1397.98	193.62	8.40
13	1373.50	183.28	8.40

**Table 13:** *SFC* parameter statistics.

Number	Original	2	8	13
*SFC*	155.74	145.73	141.69	140.69

## Data Availability

The original contributions presented in this study are included in the article. Further inquiries can be directed to the corresponding authors.

## Abbreviations

The following abbreviations are used in this manuscript:

P-TPMS	Primitive-type Triply Periodic Minimal Surface
TPMS	Triply Periodic Minimal Surface
BBD	Box–Behnken Design
*HIC*	Head Injury Criterion
*PLA*	Peak Linear Acceleration
EPS	Expanded Polystyrene
*ICP*	Intracranial Pressure
*MPS*	Maximum Principal Strain
mTBI	Mild Traumatic Brain Injury
TBI	Traumatic Brain Injury
*SFC*	Skull Fracture Correlate
AIS2+	Abbreviated Injury Scale score of 2 or greater
